# Neuroenergetics and “General Intelligence”: A Systems Biology Perspective

**DOI:** 10.3390/jintelligence8030031

**Published:** 2020-08-26

**Authors:** Tobias Debatin

**Affiliations:** Department of Educational Sciences, University of Regensburg, 93053 Regensburg, Germany; tobias.debatin@ur.de

**Keywords:** general intelligence, mitochondria, energy metabolism, network models, development of intelligence, glucose regulation, neuroenergetics, systems

## Abstract

David C. Geary proposed the efficiency of mitochondrial processes, especially the production of energy, as the most fundamental biological mechanism contributing to individual differences in general intelligence (*g*). While the efficiency of mitochondrial functioning is undoubtedly an important and highly interesting factor, I outline several reasons why other main factors of neuroenergetics should not be neglected and why a systems biology perspective should be adopted. There are many advantages for research on intelligence to focus on individual differences in the capability of the overall brain metabolism system to produce the energy currency adenosine triphosphate (ATP): higher predictive strength than single mechanisms, diverse possibilities for experimental manipulation, measurement with existing techniques and answers to unresolved questions because of multiple realizability. Many of these aspects are especially important for research on developmental processes and the building and refining of brain networks for adaptation. Focusing too much on single parts of the system, like the efficiency of mitochondrial functioning, carries the danger of missing important information about the role of neuroenergetics in intelligence and valuable research opportunities.

## 1. Introduction

As Charles E. Spearman first reported more than a century ago (Spearman 1904), cognitive tasks of all sorts are correlated; a phenomenon replicated countless times and called positive manifold. A common explanation is to assume a general intelligence (*g*) factor that leads to the positive correlations between all kinds of cognitive tasks. However, the nature of this *g* factor and whether it exists at all has been the subject of intense debate over many decades.

In this long-running debate, David C. Geary recently suggested a multi-layer theory of *g* (Geary 2018, 2019a). At the core of the different layers, he situates mitochondria and their functions, especially their efficiency of energy production (the term energy denotes the universal chemical energy currency adenosine triphosphate (ATP) and I will use the two terms interchangeably throughout this article). Mitochondria are small organelles in cells that play a very central role in cellular energy production outside and within the brain. There is no activity in the brain without energy. Therefore, all other layers of his theory, like intra- and intermodular brain networks, are dependent on the functioning of the deepest layer. 

While I mostly agree with his multi-layer structure and fully agree with the importance of individual differences in energy production for intelligence research, I do not agree with virtually equating cellular energy production with the efficiency of mitochondrial energy production as his articles strongly suggest (he also uses the labels “cellular energy production and functioning” and “efficiency of mitochondrial energy production” interchangeably for the core layer in different publications (Geary 2018, 2019b)). 

The main aim and new contribution of this article is to clarify the high relevance of other factors of neuroenergetics and to explain why I prefer a systems biology perspective (Kitano 2002) with a focus on the production of the energy currency ATP as an indicator of the capacity of the overall brain metabolism system instead of focusing on parts of the system (like the efficiency of mitochondrial functioning). For this purpose, I will first outline what the other main factors of neuroenergetics are and why they are no less fundamental than mitochondrial functioning. Then I will introduce the systems biology perspective and argue why in neuroenergetics (and in general), there should not be too much focus on single mechanisms. Finally, I will present the advantages for intelligence research to adopt this perspective and discuss implications for developmental processes and suggestions for future research. 

## 2. Main Factors of Neuroenergetics

As reviewed by Geary (2018), there is fascinating research on the relation between mitochondria and cognition; for example, Hara et al. (2014) found that rhesus apes with poor working memory skills had larger numbers of malformed mitochondria than better-performing apes. Concerning humans, Inczedy-Farkas et al. (2014) found that patients with mutations of mitochondrial DNA showed cognitive deficits compared to healthy controls. Taken together, the review of Geary clearly indicates that individual differences in the efficiency of mitochondrial functioning, especially the production of (cellular) energy, are indeed highly interesting for research on intelligence. 

Although Geary briefly mentions that not only the efficiency of mitochondrial functioning plays a role in neuroenergetics, his exclusive focus on mitochondria creates the impression that the efficiency of mitochondrial functioning and neuroenergetics are close to interchangeable. However, in the field of neuroenergetics and energy metabolism, there is also a lot of research about the mechanisms outside of mitochondria to meet the energy demands of active neurons, including the substrate supply mechanisms (Iadecola 2017; Pellerin and Magistretti 2004; Shulman et al. 2001). Metaphorically, the substrate supply mechanisms are the complex mechanisms that provide the “fuel” (e.g., the substrate glucose) to the “main power plants” (mitochondria). The importance of this becomes obvious when one considers that the efficiency of power plants is irrelevant if no fuel is available in times of high energy demand. In this case, a less efficient power plant with a constant fuel supply would be preferable. Accordingly, Lord et al. (2013) used two separate sections, “mitochondrial performance” and “glucose supply”, when discussing the energy demands of brain network oscillations. For reasons outlined below, I propose to use the more general term substrate supply instead of glucose supply. Additionally, there is a lot of research about the high importance of non-oxidative energy (ATP) generation in the active brain via a process called glycolysis, which takes place outside of mitochondria (Díaz-García et al. 2017; Fox et al. 1988; Tourigny et al. 2019). To use a metaphor again: besides providing a kind of “preprocessing” of the “fuel” glucose, glycolysis can also act as an alternative “power plant” in times of high energy demand. Therefore, when classifying the main factors of neuroenergetics, I suggest the separation should be at least threefold: mitochondrial performance, substrate supply, and glycolysis. Substrate supply and glycolysis are as fundamental for neuroenergetics as mitochondrial processes and allow for a wide range of additional individual differences.

### 2.1. Substrate Supply Mechanisms

The main energy substrates in the brain are glucose and oxygen. To fulfil its role as one of the main substrates for energy production, glucose must first pass the blood–brain barrier into the brain. A transport protein, glucose transporter 1 (GLUT1), enables this transition. Malfunctions concerning GLUT1, as in the GLUT1 deficiency syndrome, are, for example, associated with developmental delay and movement disorders (Leen et al. 2010). It was also found that the degree of impaired glucose transport is strongly related to the severity of mental retardation (Leen et al. 2010).

As there are almost no energy reserves in the brain, tight coupling between energy-consuming brain activity and substrate supply is needed. When a brain area becomes active, there is an increase in cerebral blood flow in this area; a well-known phenomenon called neurovascular coupling (for a recent review, see (Iadecola 2017)). This increase in blood flow supplies the needed additional glucose and oxygen as both are contained in the blood. Already in this mechanism are probably plenty of possibilities for individual differences. One piece of this puzzle involves individual differences in baseline metabolic rates and oxygen levels (Bulte et al. 2012; Gauthier and Hoge 2012), which are not covered via standard functional magnetic resonance imaging (fMRI) but influence the underlying blood-oxygen-level-dependent (BOLD) signal (for an introduction into standard imaging techniques and how well they are suited to assess energy metabolism see Debatin (2019)).

There is also a long tradition of investigating whether glucose and oxygen administration can improve cognitive performance via the resulting increased substrate availability, but the findings are not clear-cut (e.g., Chung et al. 2004; Hoyland et al. 2008; Messier 2004). In the case of glucose, one of the difficulties of such studies are the mechanisms of the blood–brain barrier as the amount of glucose in the blood is not directly indicative of glucose available in the brain. Therefore, for example, having a sugar drink may not increase availability in the brain. Nonetheless, positive effects have been shown frequently, but glucose and oxygen administration seem to be more effective for tasks with a high cognitive load (Owen and Sunram-Lea 2011). Furthermore, glucose administration seems to be more beneficial for persons with rather poor glucose regulation (Lamport et al. 2009). The quality of glucose regulation seems to be not only a moderator of the beneficial effects of glucose administration, but better glucose regulation is also directly associated with improved cognitive performance (Lamport et al. 2009). At that point, I want to highlight that glucose and oxygen are not the only ATP enhancing metabolic agents that can facilitate cognitive performance as beneficial effects have also been shown for pyruvate, creatine, and L-carnitine (Owen and Sunram-Lea 2011). 

Another substrate for which, despite many years of controversy, research has shown an important role in energy metabolism, is lactate (Magistretti and Allaman 2015). Under certain conditions, lactate can be derived from glucose via glycolysis. While lactate supply mechanisms would fit the topic of this section, the mechanisms are (partly) still controversial and cannot be discussed outside the context of glycolysis. Therefore, the topic will be included in the next section. 

### 2.2. Glycolysis (and Astrocytes)

When glucose has crossed the blood–brain barrier via GLUT1, within the brain, it is taken up by neurons and astrocytes via GLUT1 and GLUT3, respectively (e.g., Pellerin and Magistretti 2004). Most of this glucose is metabolized with the following order of events: first glucose is metabolized via the steps of glycolysis into pyruvate, which also leads to the formation of 2 ATP. For glycolysis, no oxygen is needed. Pyruvate then enters mitochondria and is metabolized via the tricarboxylic acid (TCA) cycle and oxidative phosphorylation, consuming oxygen (Magistretti and Allaman 2015). Complete oxidation produces a relatively large amount of 30–36 ATP. This is the main pathway for energy generation in the brain. However, during activity and in response to cognitive tasks (as opposed to the “resting brain,” which already has high energy demands), there occurs an uncoupling of glucose metabolism from oxygen consumption: there is excess non-oxidative metabolism of glucose despite the presence of oxygen (Fox et al. 1988; Shulman et al. 2001; Goyal et al. 2014). There is still controversy about what exactly is happening, but the two main streams of explanation agree that during activity, the standard process of oxidization of glucose is not able to satisfy the acute energy demands of the active cortex (Tourigny et al. 2019; Magistretti and Allaman 2015). Some even suggest that glycolysis is the main metabolic pathway of the active cortex (Tourigny et al. 2019).

Magistretti and Allaman (2015) assume (and have partly confirmed) a tight interplay between neurons and astrocytes in fulfilling energy demands during activity. Roughly spoken, neurons are predominantly oxidative, while glycolysis is predominant in astrocytes (Magistretti and Allaman 2015). In their Astrocyte-Neuron Lactate Shuttle Theory (Pellerin and Magistretti 1994), the research group assumes that activity triggers glycolysis in astrocytes, leading in several steps to the production of lactate. Lactate is then transferred to neurons that can use it as an alternative substrate for energy generation in mitochondria. 

However, the theory remains controversial. For example, Lundgaard et al. (2015) found that stimulation increases neuronal but not astrocytic glucose uptake. More specifically, neuronal stimulation seems to increase neuronal glycolysis but not lactate metabolism (Díaz-García et al. 2017). Tourigny et al. (2019) found experimental evidence indicating that both the non-oxidative supply of ATP from glycolysis as well as oxidative supply play an important role in sustaining synaptic activity and corresponding network activity. Despite its importance for the active brain, glycolysis still seems to be an underestimated feature of brain metabolism (Magistretti and Allaman 2015). The reason is probably that it accounts for only about 10% of brain glucose metabolism in adults. However, during development, glycolysis accounts for up to 30% of brain metabolism, and in some brain areas, it still accounts for up to 25% of glucose utilization in adults even in the “resting” brain ( Goyal et al. 2014; Magistretti and Allaman 2015). Overall, there is a lot of evidence indicating the crucial role of glycolysis in brain energy metabolism, especially during times of high energy demands.

## 3. The Systems Biology Perspective

Systems biology shifted the focus from characteristics of isolated parts to the overall structure and dynamics of biological systems (Kitano 2002; Klosik et al. 2017). Nonetheless, the field is often concerned with certain subsystems like the metabolic network. However, these subsystems are large and complex networks, so the different research aim is clearly visible. Regarding the classification of main factors in neuroenergetics from the last section, a systems biologist could say: okay, you intersected the brain metabolism system in relevant components or subsystems. This is, at best, a first step in understanding the complete system, especially as no detailed interdependencies between these factors were discussed, and many other factors are missing. It might not even help much in estimating the overall functionality when knowing that some components are working well (translating to small effect sizes when considering single mechanisms). 

Luckily, for many interesting purposes in intelligence research, we do not need to know all the biological details but can refer to a rather simple measure of the functionality of an overall system: its so-called carrying capacity. The term has a long history, but I consider my following definition to be within the mainstream (similar to Van Geert 1991): the maximal stable level of a certain outcome a system can provide. This maximal capacity is dependent on the resources available in the system. In ecology, this might be the maximum population size a certain environment can sustain, given its resources. In neuroenergetics it could be the maximal level of ATP the energy metabolism system can provide in times of high demand (There is fascinating research, showing that in case of high energy demand, which surpasses the production capacity, neural activity is downregulated to prevent critically low ATP levels (Ames 2000)).

The concept of carrying capacity also helps to illustrate why multiple realizability likely is a feature of many (biological) systems, including brain energy metabolism. Let us consider a sports example: we might be interested in the maximal height a high jumper can consistently surpass. In such a case, the (complex) system is the high jumper, and the carrying capacity is the maximal amount of height that can be surpassed. As can be seen from the (recent) history of high jump, there are rather small high jumpers with a long training history and taller high jumpers with a much shorter training history that have very similar high jump skills (concerning the criterion; the example is referring to Stefan Holm and Donald Thomas for those who are interested). 

So structurally different systems can lead to very similar outcomes. This has especially important implications for research on genes: twin studies of high jumpers would probably find rather high heritability estimates, but the factors leading to high jump skill might be very different. Maybe some high jumpers have genes that help them to bear the stress of many training hours while others simply have other advantageous muscle characteristics. So no obvious “high-jump genes” might be discoverable, and effect sizes might be much smaller than from twin studies despite clear genetic effects. Findings on genetics and intelligence show exactly such a pattern. In biology, there is a similar concept called “degeneracy”, which describes the findings that different structures can have very similar consequences and function, which is likely important for robustness and might play an important role in evolution (Edelman and Gally 2001).

In summary, the maximal level of ATP the brain energy metabolism system can provide is a highly interesting variable for research on intelligence and learning (Debatin 2019). This variable is the outcome of a complex system, and it seems very likely that different systems might be able to produce very similar energy production capacities.

## 4. Advantages of Focusing on ATP Production Capacity

Certainly, I do not want to give the impression that detailed research on single factors of neuroenergetics (like the efficiency of mitochondrial functioning) and especially their interplay with other factors, is unnecessary in intelligence research. However, my suggestion to focus on maximal ATP production capacity as an indicator of the functionality of the overall brain metabolism system has, in my opinion, advantages in several areas. 

First, our knowledge about the complete metabolism system is still very limited, and therefore we do not know how strongly (or if) manipulation of a specific (sub)factor affects the overall capacity of the system. There is the risk of conducting difficult biological research and finding almost no or very small effects. ATP production capacity is an indicator of the functionality of the complete system and should consequently provide larger effect sizes. This includes that there is no risk of missing “bottlenecks”: when measuring the efficiency of only a part of the system, it is possible that the overall system is limited by another factor despite the rather high functionality of a certain part. As discussed in the previous section, similarly functional systems might have different bottlenecks, and performance in energy production might be under- or overestimated by not considering the complete system. Currently, the evidence supports the assumption of larger effect sizes, as relations between levels of ATP production and cognitive performance are quite well established (also in non-clinical samples (Debatin 2019)), whereas the relation between the efficiency of mitochondrial functioning and cognition seems less established and relies more on clinical samples and subjects with severe deficiencies. According to the systems perspective, clinical findings regarding the efficicency of mitochondrial functioning might not be transferable to the general population, since severe deficiencies in a single factor are a very strong bottleneck (that might clearly influence certain outcomes), but less extreme deficiencies could be (at least partly) compensated in the overall system of all factors involved. Of course, the less reliable relationship may also be due to the difficulties in measuring mitochondrial function, but from a system perspective, a less reliable relationship is generally expected.

Second, a pragmatic advantage is that existing and relatively widely available techniques like fMRI and positron emission tomography (PET) can be used for measuring ATP production (for an overview how well standard imaging techniques are suited to assess energy metabolism see Debatin (2019); note, standard fMRI is not well suited). However, adaptive testing should be used to measure the maximal ATP production capacity: participants should be subjectively highly challenged with tasks that require as little previous knowledge as possible, since the energy expenditure depends on prior knowledge and familiarity. High subjective difficulty should reduce the familiarity problem (as even experienced individuals have to invest high effort in many tasks) as well as motivational issues. Additionally, several different tasks should ideally be used for assessment.

Third, focusing on energy expenditure clearly shows how research from related areas can be used to test the hypothesis that higher ATP levels are beneficial for cognitive performance and learning. For example, anodal transcranial direct current stimulation can improve cognitive performance, probably by its effect of increasing cortical excitability (Simonsmeier et al. 2018) and possible induction of ATP production (Rae et al. 2013). Interestingly, this effect is largest when stimulation occurs already during a learning phase, a fact I will discuss later. Furthermore, all kinds of ATP-enhancing interventions are of interest (Owen and Sunram-Lea 2011).

## 5. Energy Metabolism, Health, and Cognition

Finally, I would like to comment on Geary’s second important proposal, namely that mitochondria play a central role in explaining the link between health and cognition. As discussed in detail by Geary (2018), the efficiciency of mitochondrial energy production and other functions of mitochondria are not only linked to cognitive abilities but also to health outcomes. Additionally, as the efficiciency of mitochondrial functioning declines during aging, he suggests it is also a plausible factor in explaining cognitive and health declines during aging. Mitochondria are very similar in different cell lines and parts of the body as they derive from the same initial pool (Geary 2018), which points to a role of mitochondria in explaining the links between intelligence, health and aging.

However, in light of the previous discussion, two aspects are especially noteworthy. I agree that mitochondria might provide such a link, but I expect the link to be rather weak. One reason is that, as discussed before, mitochondrial functioning is not to be equated with neuroenergetics but is only one part of the brain energy metabolism system. While mitochondria may be similar in different parts of the body, many other important parts of the brain energy system like the substrate supply mechanisms are clearly brain-specific. Therefore, I do not see a strong association between the brain energy metabolism and metabolism outside the brain. Another possible reason for a rather weak link between mitochondria, health and cognition, which also applies to the other parts of his work, is the focus on the efficiency of mitochondrial function. With that, he seems to denote a higher ATP production per glucose molecule (Geary 2018, p. 1033). For example, it is not discussed whether a high number of mitochondria with medium efficiency is more or less beneficial for cognitive performance and health compared to a lower number of mitochondria with high efficiency. From the perspective of ATP production, the output could be very similar and not affect cognitive performance, whereas there may be long-term health benefits when mitochondria show higher efficiency.

## 6. Discussion

As Geary (2018, 2019a) noted in his mitochondrial theory of *g*, mitochondria have several important functions, but their functions concerning energy production are especially relevant for intelligence research. However, as outlined in this article, in energy production, respectively neuroenergetics, the efficiency of mitochondrial functioning is only one part of the energy metabolism system in the brain, which seems to be not more or less fundamental than the other main factors. Therefore, instead of focusing on single parts of the system, I suggested to focus on an indicator of the overall metabolism system: the maximal level of ATP the system can provide in times of high demand and described the advantages of this approach.

Besides the findings showing that higher energy-production levels are associated with higher cognitive performance (for a review of these findings and a discussion why there is no contradiction to the (refined) neural efficiency theory, see Debatin (2019)), individual differences in ATP production also have implications for research on cognitive development and learning and, consequently, in the development of *g* (Debatin 2019; Geary 2018). In recent years, more and more evidence is emerging that psychometric (fluid) intelligence is a much weaker predictor of (academic long-term) learning than traditionally assumed: Debatin et al. (2019) provide an overwiew of eight growth curve studies that investigated correlations between intelligence and learning rates in different contexts and five of them found not even a significant relation. Additionally, the study itself also found no significant relation between intelligence and the rate of learning English as a foreign language (also when controlling for intrinsic motivation). Further, a recent meta-analysis of Peng et al. (2019) investigated the relation between fluid intelligence and reading as well as mathematics. While they found a moderate relation between fluid intelligence and reading (*r* = 0.38), as well as between fluid intelligence and mathematics (*r* = 0.41), a different picture emerged when evaluating a subset of studies with longitudinal data. The effect sizes of fluid intelligence reduced to *r* = 0.17 and *r* = 0.21 respectively when predicting later reading and math performance, while partialing out initial performance. Interestingly, previous mathematics and reading skills also predicted the development of intelligence and the effect sizes were even slightly larger than the other way around. Therefore, I believe the topic deserves renewed attention, especially as learning is a central part of definitions of intelligence. Evidence points to the conclusion that Cattell’s separation in fluid and crystallized intelligence (Cattell 1943) is important but that the psychometric tests of fluid intelligence do not measure well what was intended on the original theory level (as already noted for example by Baltes et al. 1999). For a biological indicator of a general learning capacity that can literally be “invested,” energy production levels seem to be more promising because of their role, for example, in anabolism and neural plasticity (Goyal et al. 2014). The findings of the meta-analysis of Simonsmeier et al. (2018) also point in this direction by showing that anodal transcranial direct current stimulation can improve cognitive performance, especially when stimulation occurs already during a learning phase.

However, as I think that developmental processes are best considered from the perspective of dynamic systems, I do not think that ATP production levels will be a very strong, simple predictor of learning curves. Instead, I think combinations with the mutualism model of *g* (Van der Maas et al. 2006, 2017) are more promising by providing the biological basis for a (clearly genetically influenced) general resource embedded in a larger system. This also means that I certainly do not want to equate energy production levels with *g*. They explain part of the variance of *g* and are in my view an important general resource in the development of *g*, but many other factors are involved in this dynamic process. At this point I want to note that I assume that energy metabolism also plays an important role in other areas of psychology. However, this does not automatically mean positive correlations between the constructs of these different areas. As implied by the dynamic systems perspective, which I advocate, many factors and their interaction over time influence the development of a particular attribute, but these factors can be so distinct in different areas of psychology (and from person to person) that little or no correlations might result. I also want to highlight that energy production levels are not unchangeable and can be changed by factors such as nutrition and exercise (Owen and Sunram-Lea 2011; Marques-Aleixo et al. 2012). 

Robert J. Sternberg recently proposed a theory of adaptive intelligence (Sternberg 2019) and brain energy metabolism also seems to play an important role in adaptive intelligence: in my view (derived especially from: (Friston 2010; Sternberg 1999; Van der Maas et al. 2006; Van Geert 1991; Ziegler et al. 2019)), the recent state of adaptation to a certain environment of a person is strongly influenced by all resources (internal and external) relevant for adaptation to this environment over the life span. I agree completely that the quality of this recent state of adaptation is culturally defined, and the involved skills might even be useless in another environment (Sternberg 2019). However, the resources that help successful adaptation in any environment are probably very similar between environments: on the internal and biological side, ATP production capacity, especially via the relations with neural plasticity (discussed in Debatin 2019), likely is a very important general resource for adaptation (including shaping and selection of environments by actions). On the external side, in all environments, knowledgable persons who can teach the relevant specific (or more general) skills for successful adaptation are an important resource. In this context, I consider multiple realizability again to be an important topic: a similar quality of adaptation can be achieved by different resource systems. Therefore, I consider *g*, measured with the standard batteries, to be an acceptable estimator of the recent state of adaptation in cultures where verbal and numeric skills, as well as reasoning, are often required and highly valued. The question by what kind of resources that level of adaptation was acquired by a specific person is another one. High quality of adaptation should be only a weak indicator of future adaptation to certain new environments in which important parts of the resource system lose their value. For person-specific, general adaptive intelligence internal biological resources like the functionality of the brain energy metabolism system seem to be of high relevance. Connected with this topic, energy metabolism also plays an important role in evolution (e.g., Lane et al. 2013).

The typical (neuro)biological correlates of *g* can help to understand what characterizes higher levels of functionality, respectively, state of adaptation but provide only very limited information regarding the factors that influence learning and the development of these higher levels. Therefore, neurobiological studies should shift their focus from finding correlates with *g* to finding correlates with learning and adaptive intelligence, or even better, provide experimental evidence. The maximal ATP production capacity in times of high demands is a promising variable in this search, which also provides a rather precise biological foundation on how it facilitates cognitive performance and development. 

Furthermore, the topic of neuroenergetics is, of course, not only of interest for research on intelligence. Especially for personality and educational psychology as well as cognitive psychology with constructs like working memory and attention, investigating relations with ATP production capacity could provide valuable new insights. 
